# Accurate Transfer of Individual Nanoparticles onto Single Photonic Nanostructures

**DOI:** 10.1021/acsami.2c13633

**Published:** 2022-12-20

**Authors:** Javier Redolat, María Camarena-Pérez, Amadeu Griol, Miroslavna Kovylina, Angelos Xomalis, Jeremy J. Baumberg, Alejandro Martínez, Elena Pinilla-Cienfuegos

**Affiliations:** † Nanophotonics Technology Center, Universitat Politècnica de València, ValenciaE46022, Spain; ‡ NanoPhotonics Centre, Cavendish Laboratory, Department of Physics, University of Cambridge, JJ Thompson Avenue, CambridgeCB3 0HE, U.K.; § Laboratory for Mechanics of Materials and Nanostructures, Empa, Swiss Federal Laboratories for Materials Science and Technology, Thun3602, Switzerland

**Keywords:** single nanoparticle printing, soft lithography, PDMS stamps, parallel printing, capillary assembly, plasmonic cavities

## Abstract

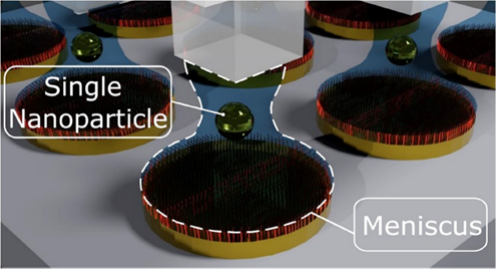

Controlled integration of metallic nanoparticles (NPs) onto photonic nanostructures enables the realization of complex devices for extreme light confinement and enhanced light–matter interaction. For instance, such NPs could be massively integrated on metal plates to build nanoparticle-on-mirror (NPoM) nanocavities or photonic integrated waveguides (WGs) to build WG-driven nanoantennas. However, metallic NPs are usually deposited via drop-casting, which prevents their accurate positioning. Here, we present a methodology for precise transfer and positioning of individual NPs onto different photonic nanostructures. Our method is based on soft lithography printing that employs elastomeric stamp-assisted transfer of individual NPs onto a single nanostructure. It can also parallel imprint many individual NPs with high throughput and accuracy in a single step. Raman spectroscopy confirms enhanced light–matter interactions in the resulting NPoM-based nanophotonic devices. Our method mixes *top-down* and *bottom-up* nanofabrication techniques and shows the potential of building complex photonic nanodevices for multiple applications ranging from enhanced sensing and spectroscopy to signal processing.

## 1 Introduction

Controlled, efficient, and accurate positioning of single metallic nanoparticles (NPs) on micro- and nanopatterned substrates is crucial for a wide range of applications such as photovoltaics,^1^ lab-on-a-chip, and biosensors,^2^ as well as large-scale plasmonic chemistry.^3^ Among the different nanophotonic structures that include NPs, nanoparticle-on-mirror (NPoM) cavities are highly interesting because they offer extreme light confinement within nm-scale gaps.^4−6^ Essentially, a NPoM (often termed patch antenna or metal–insulator–metal waveguide (WG)) is formed by depositing a metallic NP on top of a metallic mirror covered by a self-assembled molecular monolayer (SAM) so that under suitable illumination, light is confined in the nm-scale gap separating the metal surfaces. For a wide range of applications, such as absorbing elements,^7^ NPoMs are assembled over large areas so NPs are deposited on metal mirrors without requiring any position accuracy. Conventionally this can be done via drop-casting of NPs onto metallic surfaces covered by a SAM, which has become the most straightforward way to produce nm-scale cavities with high accuracy. However, some applications, such as molecular frequency upconversion,^8,9^ may require the integration of a single NP with a μm-scale antenna instead of a metal mirror.^10,11^ Here, depositing single NPs onto nanostructures with high positional accuracy is of major importance. This is because for instance focusing mid-infrared light on an antenna localizes the optical field in specific locations, and the NPoMs have to be placed in these locations to maximize nonlinear interactions with visible/near infrared light.

Current NP printing techniques developed for the controlled imprint of NPs on surfaces are based on colloidal self-assembly methods or utilizing capillary forces within polymeric templates.^12−16^ Single NP positioning onto individual lithographed nanostructures is challenging. So far, single NP positioning can be obtained via laser printing onto glass^17,18^ (not metals) or by atomic force microscopy (AFM)^19,20^ but these are complex, expensive, and slow (serial) methods.

In this work, we introduce a large-scale method for the accurate delivery of single NPs on complex photonic nanostructures (such as μm-scale metallic antennas antennas or integrated WGs) based on a stamp-assisted soft lithography method.^21^ The main advantage of soft lithography is that it is a parallel nanoprinting technique that provides high-throughput and high simplicity together with the possibility of the precise transfer of multiple individual NPs onto different planar and nonplanar nanostructures. A key point is that it can be used for both single-step positioning of multiple individual NPs onto an array of antennas as well as for the positioning of a single NP onto an individual photonic nanostructure. Our approach is cost-effective and a robust nanolithography methodology that does not require complex (and expensive) instrumentation. We validate our method by performing transfer of NPs onto different kinds of photonic structures, such as μm-scale plasmonic antennas and integrated Si_3_N_4_ WGs. In addition, we show enhanced light–matter interaction in representative resulting devices using surface-enhanced Raman spectroscopy (SERS) measurements.

## 2 Materials and Methods

### 2.1 μ-Printing Device

Manual micropositioners, 4D stage (*XYZ*θ), have a maximum travel of 13 mm at *XY* along each axis, 10 mm along the *Z* axis with submicron resolution, the goniometer (θ) is used for sample rotation. FSR: force sensitive resistor: Squared sensing area of 5 × 5 mm (thickness of 0.45 mm), actuation force 0.1 N, and sensitivity range to 10 N. For the electronic design, a Nano Arduino based on the ATmega328P microcontroller is used. Optical microscope system, Navitar 6.5× zoom combination system (O1) specifications: lens attach 1.5× + prime lens 6.5× zoom + adapter 2.0×. Working distance: 51 mm; system magnification (low/high) 2.1–13.50; NA objective (low/high): 0.034–0.106; resolve limit (μm) (low/high): 9.8–3.14; matching pixel size (μm) (low/high): 10.20–21.26; depth of field (low/high): 0.43–0.04.

### 2.2 Stamp and Master Fabrication

Polydimethylsiloxane (PMDS) stamps were prepared by replica molding of the fabricated master. The PDMS stamp fabrication was done employing the kit named: “Kit SiliconElastomer Sylgard 18”. Sylgard 184 is a bicomponent system for the fabrication of silicone stamps that is formed by a base and a curling agent. Once the proper mixture was prepared, the PDMS stamps were cured at 90 °C for 45 min and peeled off the master.

The silicon master, consisting of a periodic array of squared wells, was fabricated using a standard direct writing process based on electron beam lithography. The fabrication was carried out on standard silicon samples (resistivity ρ ∼ 1^–10^ W cm^–1^, with a lightly p-doping of ∼10^15^ cm^–3^). The fabrication process is based on an electron beam direct writing process performed on a coated 100 nm poly(methyl 2-methylpropenoate) (PMMA) resist film. The mentioned electron beam exposure, performed with a Raith150 tool, was optimized in order to reach the required dimensions employing an acceleration voltage of 10 KeV and an aperture size of 30 μm. After developing the PMMA resist, the resist patterns were transferred into the Silicon samples employing an optimized inductively coupled plasma-reactive ion etching process with fluoride gases (SF6 and CF4). Finally, the master was Al-coated to facilitate the peel off the PDMS stamp from the master. Otherwise, the PDMS polymer can break in the peel off step due to adhesion to the micrometric lithographed motifs. Once fabricated, the master can be reused multiple times as well as the stamps. We want to highlight that the shape of the holes in the master sample (therefore the shape of the PDMS stamp pillars) is square for technical reasons. This is because the fabrication of a master sample in silicon by ebeam lithography is more accurate, faster, and reliable for square shapes than for circular shapes.

### 2.3 Biphenyl-4-thiol Functionalization

Biphenyl-4-thiol (BPT, 97%) molecules were purchased from *Merck-Sigma Aldrich*. First, piranha solution (H_2_SO_4_/H_2_O_2_, 1:1) was used for glass cleaning. BPT SAMs were prepared by dipping the substrates in 1 mM BPT in ethanol (absolute, reagent grade) for 14 h. Finally, the sample was sonicated in ethanol for 3 min, rinsed with ethanol, and dried under N_2_ stream.

The quality of the SAMs was evaluated by advancing-receding contact angle measurements in a Ramé-hart automatized goniometer and by AFM imaging.

### 2.4 Drop Casting Au-NP Deposition on Disks

The drop casting was performed delivering a 10 μL drop of 60 nm Au-NP solution onto the Disk 3 sample, left for 5 min, and then rinsed with Milli-Q water, with a concentration of *C* = (2.3 ± 0.5) × 10^10^ particles/ml. Finally, the substrate was dried under a N_2_ stream. Water suspension of spherical citrate-capped 60 nm Au NPs was purchased from Nanopartz.

### 2.5 3-Aminopropyltriethoxysilane Functionalization

3-Aminopropyltriethoxysilane (APTES, 99%) molecules and solvents were purchased from *Merck-Sigma Aldrich* and used without previous purification. APTES SAMs were prepared by dipping the substrates in 1 mM APTES in ethanol (absolute, reagent grade) for 45 min, then rinsed with ethanol, and finally dried under a N_2_ stream.

### 2.6 Atomic Force Microscopy Imaging

Alpha300 RA (Raman-AFM) from *WITec* was employed for the AFM sample characterization. All measurements were performed in AC mode. Sharp silicon probes without coating (*K* ∼ 42 N/m, *f*_0_ ∼ 320 kHz) were purchased from PPP-NCH (Nanosensors). All AFM images were processed with WSxM software from Nanotec Electrónica S.L.^22^

### 2.7 Scanning Electron Microscopy Imaging

High-resolution field emission scanning electron microscopy (HRFESEM) was utilized for imaging the transferred NPs onto the different photonic nanostructures. Each sample was scanned with a ZEISS GeminiSEM 500 with resolution: 0.5 nm at 15 kV; 0.9 nm at 1 kV, 1.0 nm at 500 V.

## 3 Results and Discussion

**Figure 1:**
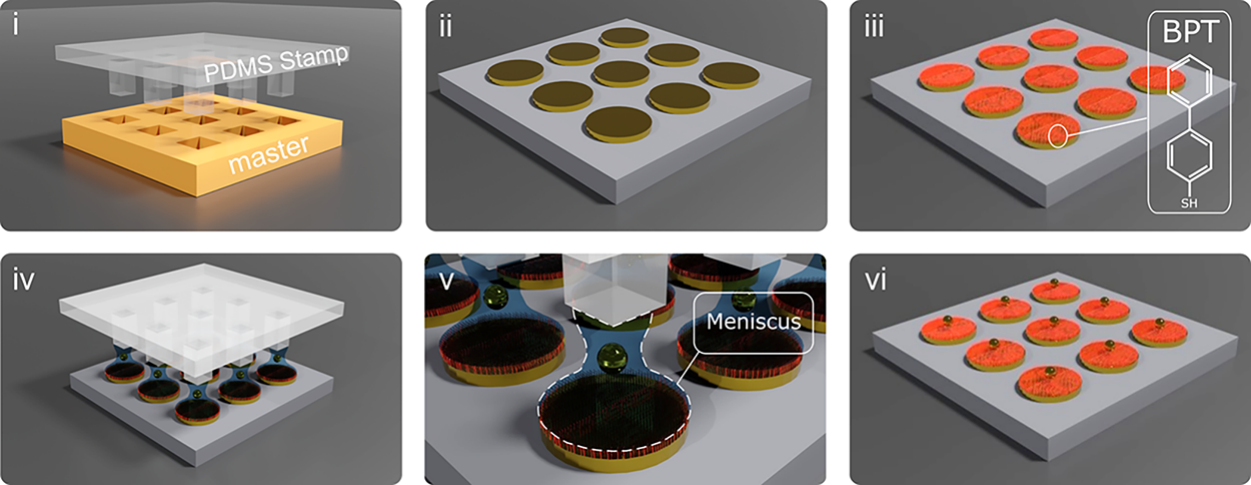
Schematic parallel printing method: (i) PDMS stamp fabrication by cast molding; (ii) fabrication of the photonic micronanostructure; (iii) sample functionalization with BPT SAM; (iv) NP printing process with localized menisci; (v) meniscus formation between the stamp protrusion and the functionalized lithographed sample. Each NP is trapped and guided by the meniscus to the antenna array; (vi) lift off PDMS stamp leaving NPs attached to the sample.

Our method (Figure 1) is based on lithographically controlled wetting (LCW) that provides a route for the in-situ fabrication of NPoM cavities based on elastomeric stamps, typically made of PDMS (Figure 1i).^23^ The PDMS stamps are designed according to the target photonic structures with the aim to position the NPs at each single antenna or WG (Figure 1ii). The LCW consists of the stamp-assisted deposition of a soluble material from a solution (or in our case, a colloidal suspension of Au NPs in water). To facilitate the delivery of the NPs, the lithographed motifs of the sample are functionalized with a self-assembled monolayer (SAM) that improves the affinity between the NP and the substrate (Figure 1iii). As the stamp is placed in contact with a colloidal suspension on a surface, the capillary forces drive the liquid to distribute only under the protrusions of the stamp producing an array of menisci (Figure 1iv). The NPs are transferred in a pattern defined by the size of the stamp pillars, with a minimum feature size as small as the meniscus formed at the stamp protrusion (i.e., pillar, Figure 1v). The NP is locally trapped in the meniscus, and due to the chemical affinity with the functionalized surface, the NP transfers to the surface. The stamp is then lifted-off and, as the solvent evaporates, individual NPs are patterned on the surface with the same length scale as that of the stamp (Figure 1vi).

The concentration of the NP colloidal suspension, the affinity between stamp–solution–substrate, and the applied pressure between stamp and the target nanostructure are parameters that can be modified to deliver different patterns of the same components without modifying the stamp features. Moreover, the flexibility of the PDMS stamp and the ability to achieve conformal, nanometric level contact between the stamp and the substrate are both advantageous for printing over nanometric and micrometric photonic antennas such as disks, bow-ties, WGs, or even curved substrates. Furthermore, the PDMS stamp shows higher hydrophobicity (contact angle ∼ 100°, see SI 1) than the functionalized substrates (contact angle of ∼ 80° for BPT-Au antennas, see SI 2) which facilitates the meniscus formation and, therefore, the suitable conditions for trapping/transferring NPs.

**Figure 2:**
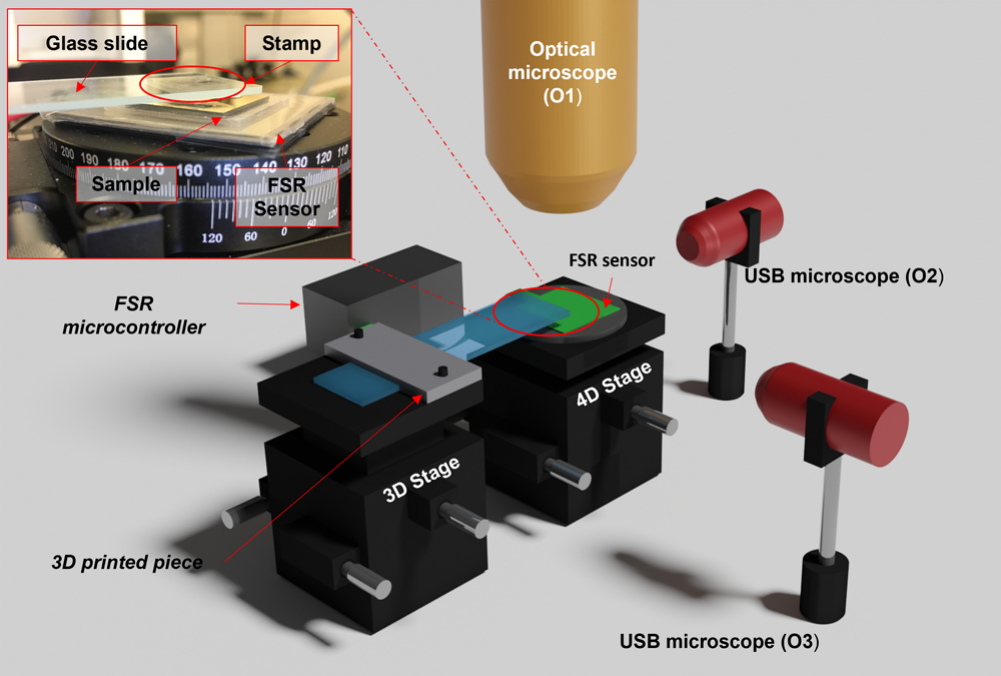
3D schematic of the nanoimprint setup. Inset shows a photo of the stamp, sample positioning, and FSR.

One of the crucial parameters required for the deposition of single NPs onto specific locations onto the antennas array is the precise alignment between the stamp and the array substrate. To provide this, a μ-printing device comprising pressure monitoring is constructed to deliver submicrometric accuracy and high reproducibility with precise control at every step of the stamping method. Here, an optical microscope (O1) is mounted on a *XYZ* translational stage allowing coarse alignment and focusing on the *z*-axis (Figure 2). O1 allows μm-scale precision with the aid of a digital camera and white illumination launched through via an optical fiber. A 4D stage (*XYZ*θ) aligned with O1, is used for the nm-scale sample positioning. A force sensitive resistor (FSR) is placed on top of the translational stage for pressure monitoring while the sample is on top. A second translational 3D stage is used to place the stamp. The PDMS stamp is mounted on a glass slide, attached with transparent sticky tape to allow monitoring from the top (O1). The glass slide is fixed to the translational stage with a 3D printed piece purpose-designed for this configuration. This custom-build holder can be screwed on the 3D stage to adapt the position of the PDMS stamp over different samples. In addition, two optical microscopes (O2 and O3) mounted on adjustable height posts allow monitoring along the *z*-axis. They are connected to a PC via USB and are situated at 90° to facilitate the correct alignment of the system stamp/substrate. They allow real time monitoring to enable feedback and quantification.

The printing experiment is performed as follows: First, 100 μL of NP solution is drop casted onto the stamp for about 2 min. A concentration of *C* = (2.3 ± 0.5) × 10^10^ particles/ml of citrate-caped 60 nm Au NPs suspended in water was used (concentration, size, and size distribution of Au-NPs were characterized by dynamic light scattering measurements, included in SI 3). Then, excess solution is removed with a tissue from the side of the stamp (not from top). Further details of stamp inking are described in SI 4. The glass slide with the “wet” stamp is then fixed to the 3D stage and rapidly aligned on top of the nanostructured sample with the micropositioning mountings. Manual alignment is optically controlled via O1. Once the stamp is correctly aligned with the sample, it is approached until it is pressed with a force of ∼2 N. With forces (*F*) below a threshold value of 1.5 to 2 N for square stamps with an area of 8 mm × 8 mm and a height of (0.48 ± 0.02) mm, no transfer occurs. Experiments with *F* > 3 N were not carried out. Higher pressures cause the stamp columns to collapse, and the NPs to spread uncontrollably across the surface. Finally, the stamp is lifted-off the sample. For the imprinting to succeed, this whole process must take less than ∼2 min, otherwise, the solvent evaporates, and the transfer yield dramatically decreases. The whole process was carried out under environmental conditions of room temperature between 23 and 25 °C and 50–55% humidity.

**Figure 3:**
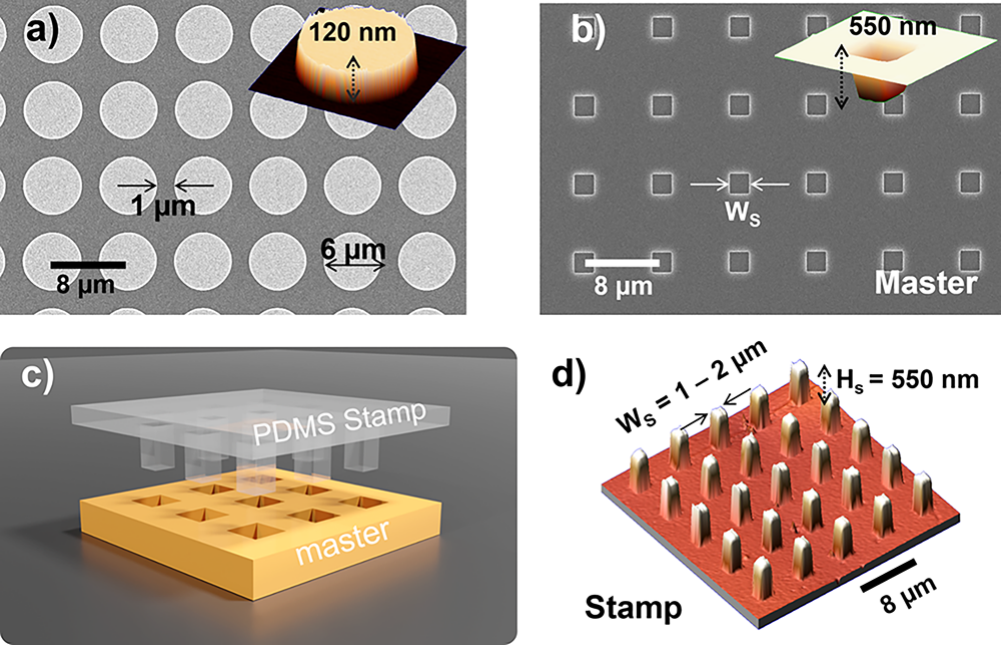
Nanoparticle transfer substrates and stamp master. (a) SEM image of Au resonator disk array of 6 μm diameter, 1 μm separation, and 8 μm pitch. Au disks are patterned onto a Si substrate. Inset shows AFM measurement of single Au disk of 120 nm height. (b) SEM image of master on Si used for the PDMS stamp molding. Hollow squares have width W1_S_ = 1 μm (Stamp 1) and W2_S_ = 2 μm (Stamp 2), and depths of 550 nm, 8 μm pitch for both. Inset shows AFM measurement of a single pit giving 550 nm depth. (c) Sketch of PDMS stamp fabrication from the Si master. (d) 3D AFM image of a fabricated PDMS stamp indicating its geometrical dimensions.

To prove that this yields a single-step large-scale nanopositioning method for the parallel transfer of individual gold NPs on multiple photonic structures in one single step, we use three samples (Disk 1, Disk 2, and Disk 3). The samples consist of 4 × 4 arrays of gold resonators disks on a silicon substrate. Each array was formed by 24 × 24 Au disk antennas (sample Disk 1 = 2304 disks) and 25 × 25 disks (samples Disk 2 and Disk 3 = 2400 disks) of diameter Φ_D_ = 6 μm, with 1 μm separation (*P* = 8 μm pitch) and 120 nm height (Figure 3a). A Si master was fabricated of identical sample dimensions for producing the polymeric stamps (see Materials and Methods section). Hollow squares were lithographically fabricated with two different widths of W1_S_ = 1 μm in the case of Stamp 1 and W2_S_ = 2 μm for Stamp 2, and depths of 550 nm, using 8 μm pitch in both cases to match the target samples (Disk 1 and Disk 2, Figure 3b). The polymeric stamps were then prepared by cast molding over the fabricated master substrates (Materials and Methods section, and (Figure 3c)). Two PDMS stamps were patterned with square relief features with the same number of columns as the patterned gold disks samples and the same pitch to ensure a perfect match. In the first case (Stamp 1), square 24 × 24 columns of side W1_S_ = 1 μm and height of *H*_S_ = 550 nm (*P* = 8 μm pitch), while the second case (Stamp 2) uses 25 × 25 columns of side W2_S_ = 2 μm and height of *H*_S_ = 550 nm (*P* = 8 μm pitch) (Figure 3d). The gold disk antenna array was functionalized with a BPT SAM for effective NP transfer in both cases. Once the sample was functionalized, spherical Au-NPs of 60 nm diameter were imprinted in a single-step.

Imprint experiments were performed with the two designed stamps and two functionalized samples of Au disk arrays (BPT-Au disks, Disk 1 and Disk 2). In addition, a regular drop-casting experiment was performed depositing the same concentration of colloidal dispersion (*C* = (2.3 ± 0.5) × 10^10^ particles/mL) directly onto a third BPT-Au disk sample (Disk 3) to compare the yield of the transfer method. We present statistics of the first experiment (Stamp 1) in Table 1, and of the second experiment (Stamp 2) in Table 2, accordingly. For each array, the number of disks with single NPs (1 NP), two (2 NP), three NPs (3 NP), and clusters (>3 NP) as well as the percentage yield (considering the total number of disks: 576 disks for each array (A1–A4) of Disk 1 sample and 625 for each array (A1–A4) of Disk 2 sample) and the total average transfer yield is shown. The counting of the NP for the transfer yield calculation was performed from high-resolution HRFESEM images (see Materials and Methods section).

**Table 1:**
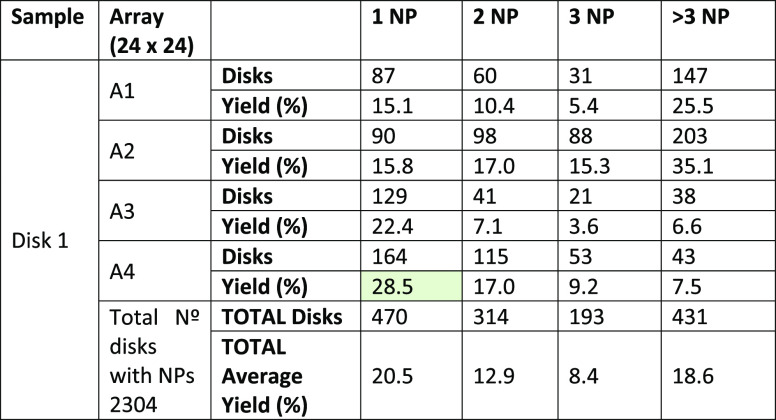
Nanoparticle Positioning Statistics for Stamp 1 (W1_S_ = 1 μm) Transfer onto Sample Disk 1^a^

a The highest yield of 28% is highlighted in green.

**Table 2:**
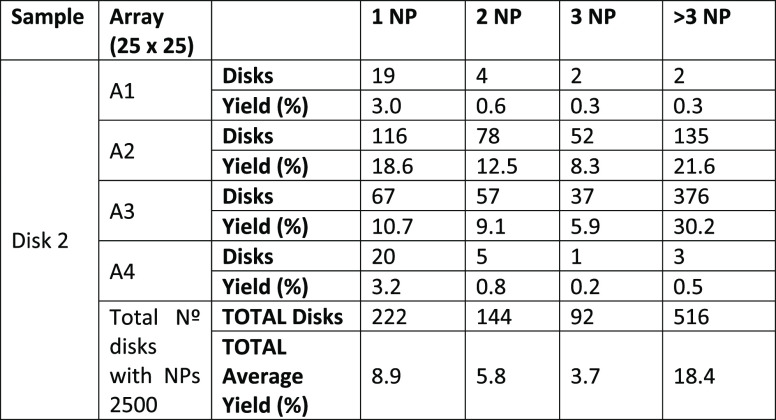
Nanoparticle Positioning Statistics for Stamp 2 (W2_S_ = 2 μm) Transfer onto Sample Disk 2

In sample Disk 1 (with Stamp 1, W1_S_ = 550 nm height × 1 μm side columns), we find a mean stamping average yield of 20.5% for single NP transfer, while for sample Disk 2 (with Stamp 2, W2_S_ = 550 nm height × 2 μm side columns), this was found to be much smaller, 8.9% (average). As can be seen for both cases, the average transfer yield of 1 NP deposition is the highest and decreases for 2 NP and 3 NP transfers. The use of a stamp with narrower pillars (W1_S_) leads to better transfer yields for single NPs. We attribute this improvement to the fact that the meniscus formed between pillar and sample is smaller for the smaller pillars, as expected. The lower limit of meniscus size between the stamp pillar and the nanostructure is limited to the size of pillar and sample disk; therefore the nanopositioning of single 60 nm Au NPs is more efficient with the W1_S_ = 1 μm PDMS pillars.

Finally, the NP cluster limit has been considered in both cases for disks transferring more than 3 NPs. As can be seen in the tables, for both types of stamps, this value saturates to ∼ 18% (average). In this case, the clusters presented in our transfer method do not depend on the size of the pillar but on the well-known “coffee ring effect” that we observe in our disks’ perimeter at the borders of the stamp. To avoid this effect is challenging, which results in NP deposition along the perimeter of a droplet induced by capillary forces when the solution dries.^24^ Overall, we note that both cases result in much higher NP transfer yield than simple drop-casting (∼ 1%), as expected.

We would like to emphasize that these experiments are performed in a single-step and the yield can be further improved by repeating the transfer step. We find that once the NPs are transferred, they get permanently attached to the surface. To prove this, we rinse with water the printed Au NPs as well as applying sonication and immersing in piranha solution. In all cases, the NPs could not be removed from the surface, so we had to repeat the transfer without affecting the NPs that were previously printed.

Moreover, the elastomeric PDMS stamps are mechanically and chemically stable allowing reuse >50 times over several months without noticeable degradation in performance. We note that the stamp was stored in a low humidity environment and not exposed to strong acids or bases.

**Figure 4:**
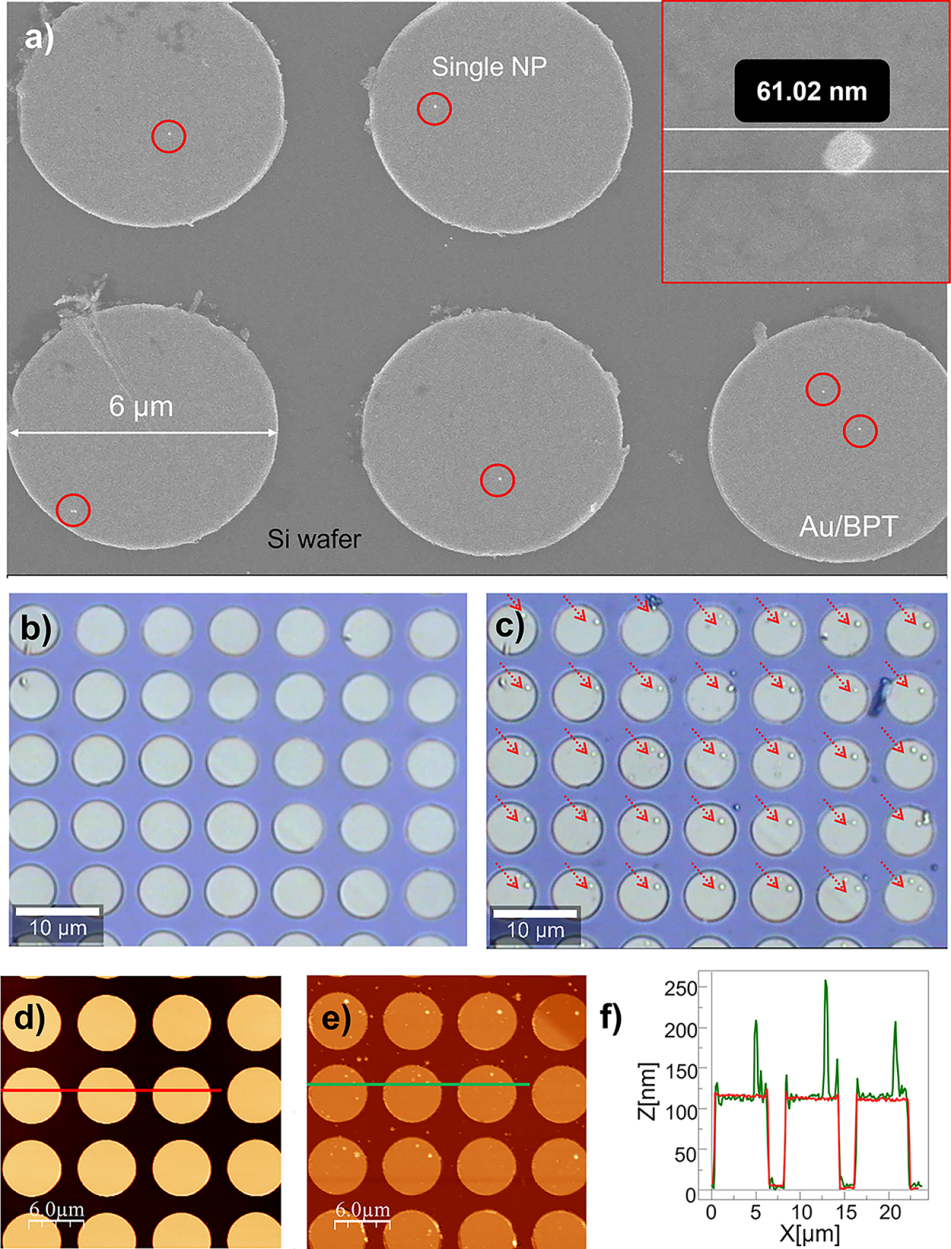
High fidelity single-step large-scale single NP transfer method. (a) SEM image of 60 nm Au NPs transferred individually with W1_S_ = 1 μm stamp. (b) Optical microscope image of Au disk array of 6 μm diameter fabricated on a Si wafer before transfer of NPs with W2_S_ = 2 μm stamp. (c) Optical microscope image of the same area of functionalized Au/BPT disks after single-step NP transfer with W2_S_ = 2 μm stamp. AFM measurements of the same area before (d) and after (e) the NP transfer. (f) AFM profile for three Au/BPT disks with single NPs printed on top.

To better quantify our single-step printing, we show large-scale printing of single NPs onto disk antenna arrays (Figure 4). Examples of single NP positioning onto the disk antennas is shown for the Disk 1 sample (Figure 4a). An area of 7 × 5 Au disks from the Disk 2 sample is shown before and after the NP transfer (Figure 4b,c). Every disk contains a single NP placed in the same position over the disk (marked with red arrows). Only in few cases, we find two NP transfers in one disk. To confirm the positioning and size of the transferred NPs, we show AFM images of Disk 2 before and after the transfer (Figure 4d–f). The same type of experiment was carried out for positioning bigger Au NP (150 nm spherical citrate-capped Au NPs suspended in water). The 150 nm NPs were successfully transferred onto BPT functionalized samples of Au disk arrays using W1_S_ = 1 μm stamp (see SI 6).

It is worth mentioning that the location of NPs inside the disks influences the performance of the NPoM structure. The spatial dependence of SERS, for increasing radial positions (*r*) of NPs from the disk center is studied in Figure 3 from reference (10). The Au disk supports high-order modes in the visible range resulting in near-field standing waves. When a NP is placed on electric-field antinodes, its SERS intensity is boosted. On the contrary, when NPs are placed on electric-field nodes then no enhancement of SERS is expected (similar strength to NPoMs).

**Figure 5:**
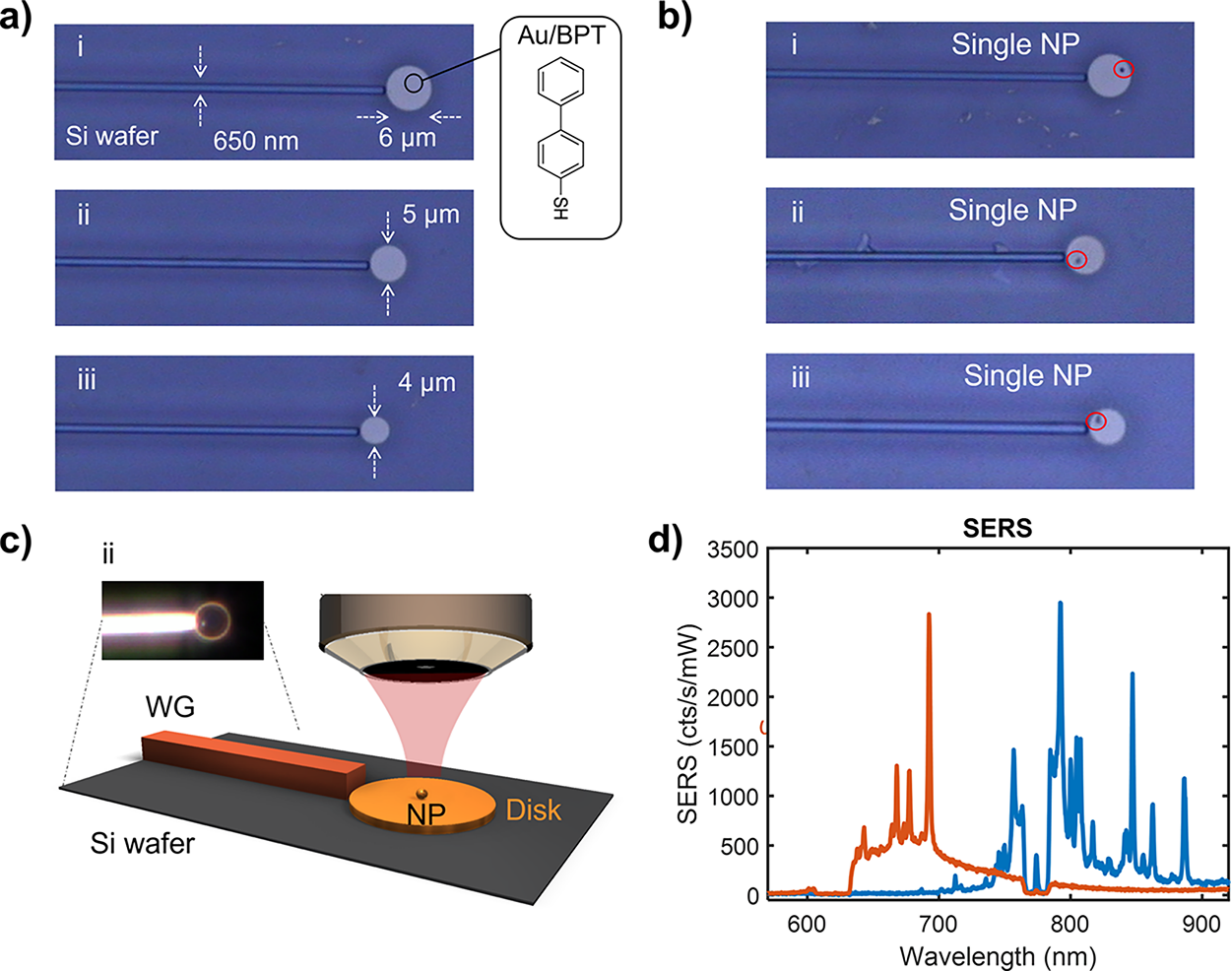
Single NP stamping onto NPoRs coupled with standard Si_3_N_4_ WGs. (a) Optical microscopy images of BPT-functionalized Au disks of different diameters: (i): 6 μm, (ii): 5 μm, and (iii): 4 μm at the end of 650 nm wide Si_3_N_4_ WGs. (b) Optical microscopy images of same structures with NPs of 60 nm diameter printed on top. (c) Scheme of NPoR interfacing a WG and free-space SERS characterization. Inset shows dark-field image of case ii. (d) BPT SERS spectrum for pump wavelengths of 633 nm (red) and 785 nm (blue).

It is possible using the μ-printing device to laterally control the stamp onto nanometric structures within 650 nm (submicrometric resolution), so as to be able to deliver single NPs onto individual structures. This evidences the capability of controlled single NP printing onto different complex nanostructures. To apply this transfer to standard photonic devices, we show the transfer method onto a metallic μm-scale disk antenna fabricated interfacing a Si_3_N_4_ WG. This photonic structure has been proposed as a promising platform for on-chip SERS sensing^9^ as it delivers high in- and out-coupling signal efficiencies. The aim here is to place a single NP on the disk antenna to form a so-called nanoparticle-on-resonator (NPoR)^10^ design, with the WG used to couple light efficiently in and out of the nanocavity. Using the same transfer strategy, we deliver single NPs onto Au disks at the WG end, for several disk diameters: (i) 6 μm, (ii) 5 μm, and (iii) 4 μm (Figure 5a). In this case, stamps with 1 μm × 1 μm pillars were used to form smaller menisci and give better yields. Single 60 nm Au-NPs were successfully positioned on all the Au/BPT functionalized disks after two consecutive transfers without disturbing the previously printed NPs (Figure 5b). To validate the presence of the BPT-SAM in the fabricated NPoR photonic structure, we performed SERS experiments with free-space excitation and collection from above (Figure 5c). The SERS spectrum of BPT (case ii of 5 μm disk) is obtained (Figure 5d) for pump wavelengths of 633 nm (red) and 785 nm (blue) which shows enhanced vibrational BPT signatures due to the elevated near-field of the NPoR geometry. Given that our NP transfer method creates nanocavities on Au disks, we expect similar SERS signal efficiencies as for the NPoR antenna.

**Figure 6:**
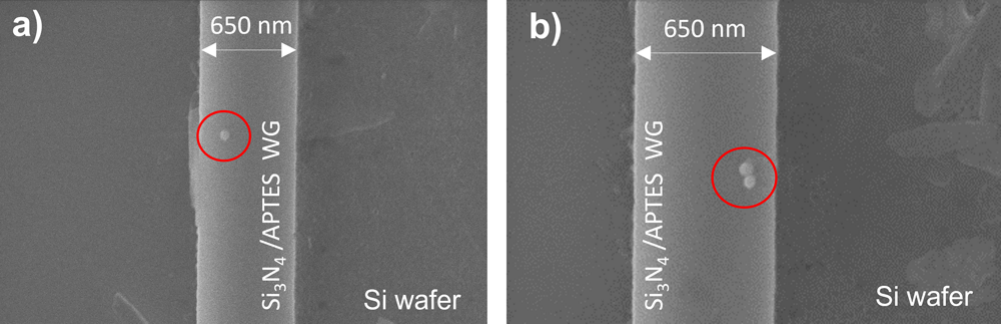
Single NP transfer onto Si_3_N_4_ WGs. SEM image of (a) single and (b) double Au NPs transferred onto a Si_3_N_4_ WG prefunctionalized with APTES.

Further, single Au NP positioning was also achieved onto Si_3_N_4_ WGs functionalized with APTES (Figure 6a,b). Although the formation of water meniscus between PDMS stamps and APTES functionalized surfaces is known to be chemically stable (even for NP solutions at different PH values),^25,26^ in these cases, the transfer was found to be more challenging. Not only for the difficulty in the stamp alignment but also because the meniscus formation is less stable in this type of nanostructures (nanometric WGs in comparison with micrometric round-shaped antennas), from the mechanical point of view. In this sense, if the stamp is not perfectly aligned, the meniscus cannot be formed or is less stable if the stamp is tilted or shifted from the WG position. However, after two or three transfer repeats, single NP transfer is achieved in all cases.

Finally, it is important to note that by switching from manual micropositioners to motorized stages for a more precise stamp-sample alignment, the method’s success rate could be increased. It is also possible to modify the method to reach higher rates by including an automated syringe under the microscope to control the amount of colloidal fluid placed onto the stamp and carrying out the entire procedure in a cleanroom to prevent temperature changes and contamination.

## 4 Conclusions

We developed a reproducible, single-step, and cost-effective method for the controlled nanopositioning of single NPs for both parallel printing and single positioning of individual NPs onto standard lithographically fabricated photonic nanostructures with submicron accuracy in a single step. Taking advantage of the capillary forces in elastomeric stamps and utilizing a custom-built μ-positioning device, we achieve a single-step NP transfer yield of up to 28%. In addition, the methodology is utilized to transfer NPs to more complex photonic structure geometries such as metallic disk antennae and integrated WGs, improving not only the drop-casting yield but also gaining control of the NP positioning over the micronanostructures. We believe this process can be applied to NPs of other materials (such as metal oxides or polymers) as long as the suspension of the NPs does not contain solvents affecting the chemical stability of the PDMS stamp and the surfaces are functionalized with SAMs. This large-scale approach paves the way toward deterministic positioning of individual NPs for a wide range of applications including NPoM fabricated cavities on nanophotonic structures for advanced spectroscopic architectures on-a-chip.

## Supporting Information Available

Water contact angle measurement of the PDMS stamp; advancing and receding water contact angle measurements of the Au antennas substrate before and after BPT functionalization; dynamic light scattering measurements of Au-NP colloidal suspension; stamp inking procedure; meniscus formation; transfer of individual 150 nm Au NPs onto functionalized nanoantennas (PDF)
