# Supplementary material for: Accurate Transfer of Individual Nanoparticles onto Single Photonic Nanostructures
Source: ACS Appl Mater Interfaces. 2022 Dec 20;15(2):3558–65. doi: 10.1021/acsami.2c13633 (PMC9869328; doi:10.1021/acsami.2c13633)
Supplement: Supplementary file 1 — am2c13633_si_001.pdf [file am2c13633_si_001.pdf]

SUPPORTING INFORMATION

ACCURATE TRANSFER OF INDIVIDUAL NANOPARTICLES ONTO SINGLE PHOTONIC NANOSTRUCTURES

Javier Redolat[1], María Camarena-Pérez[1], Amadeu Griol[1], Miroslavna Kovylina[1], Angelos Xomalis[2,3], Jeremy J. Baumberg[2],
Alejandro Martínez [1*] and Elena Pinilla-Cienfuegos [1*]

[1]Nanophotonics Technology Center, Universitat Politècnica de València, Valencia E46022, Spain
[2]NanoPhotonics Centre, Cavendish Laboratory, Department of Physics, JJ Thompson Avenue, University of Cambridge, Cambridge CB3 0HE, United Kingdom
[3]Empa, Swiss Federal Laboratories for Materials Science and Technology, Laboratory for Mechanics of Materials and Nanostructures, Thun, 3602, Switzerland

*Corresponding authors: Elena Pinilla Cienfuegos (epinilla@ntc.upv.es) and Alejandro Martínez (amartinez@ntc.upv.es)

S1. Water contact angle measurements of PDMS.

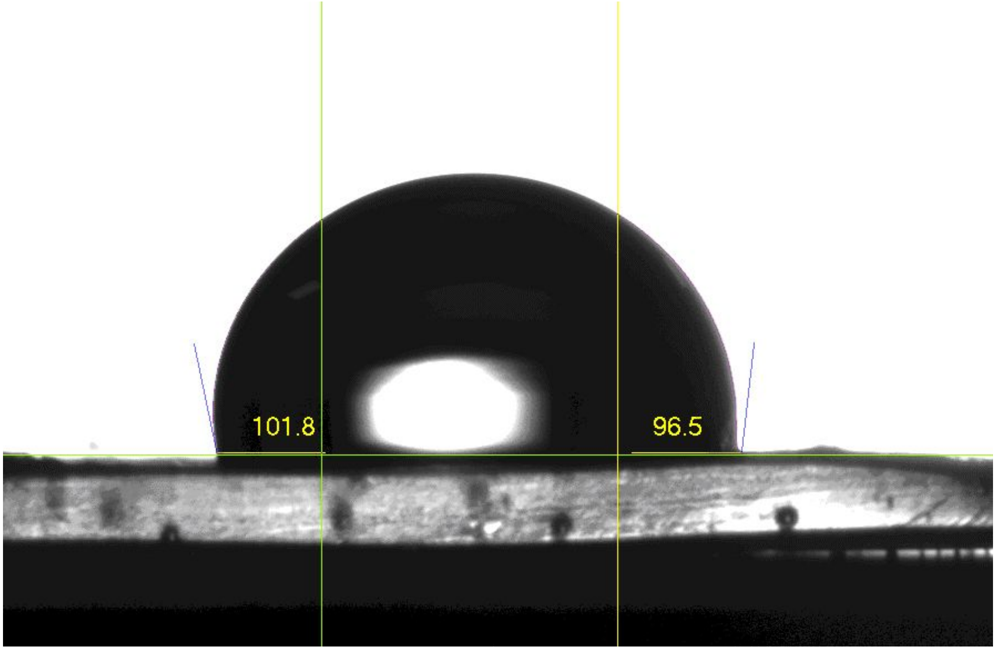

Figure S1. ***Water contact angle measurement****. Silhouette of ultrapure H2O drop laid on the surface of a PDMS stamp.*

S2. Water contact angle measurements of BPT-Au antennas.

Advancing and receding water contact angle measurements were performed by the "add and remove volum" method in a Ramé-hart Model 90 Standard Goniometer with

Dropimage Standard software, equipped with an automated dispensing system. This device includes software as well as a led illuminator, 3-axis levelling stage, digital camera, microsyringe fixture and assembly for manual dispensing. The system is improved with an automated dispensing system and manual tilting base.

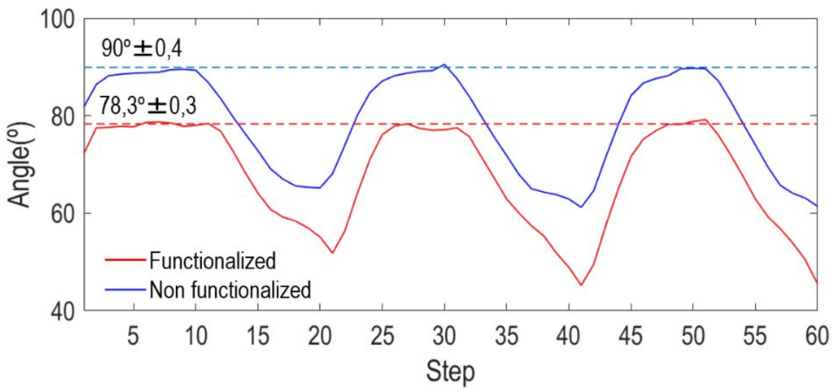

Figure S2. ***Advancing/receding contact angle cycles:*** *performed on Au-functionalized BPT antenna (red line) and non-functionalized gold sample (blue line).*

S3. Dynamic Light Scattering (DLS) measurements of Au-NPs colloidal suspension

The concentration and size of the citrate-capped Au-NPs in water solution have been evaluated by Dynamic Light Scattering (DLS) measurements (Figure S3).

We found a concentration of C = $(2.3 \pm 0.5) \times 10^{10}$ particles/ml and NP Mean Size of (61.9 ± 1.7) nm (Table 1).

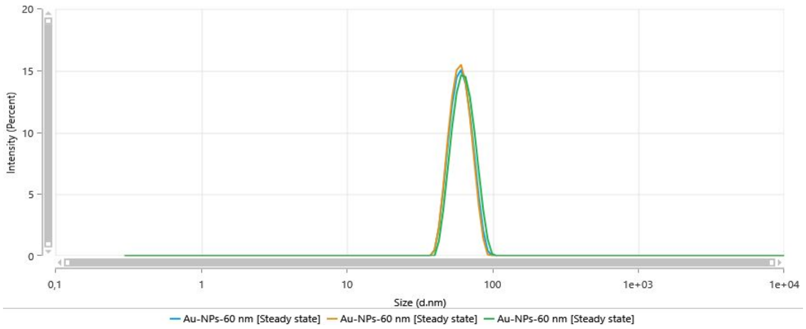

Figure S3. Dynamic Light Scattering (DLS) measurements of size dispersion of Au-NPs.

**Name**	**mean**	**Standard deviation**	**RSD**	**Min**	**Max**
**Peak One Area by Number (%)**	100	0	0	100	100
**Peak One Mean by Number (nm)**	53,28	1,225	2,3	52,53	54,7
**Peak One Width by Number (nm)**	8,624	0,2895	3,357	8,361	8,934
**Total Number Concentration (particles/ml)**	2,30E+10	4,58E+09	19,89	1,79E+10	2,66E+10
**Peak One Area by Intensity (%)**	100	0	0	100	100
**Peak One Mean by Intensity (nm)**	61,96	1,708	2,756	60,67	63,89
**Peak One Width by Intensity (nm)**	10,45	0,5387	5,154	9,943	11,02

TABLE S1. Concentration, size, and size distribution of Au-NPs by Dynamic Light Scattering measurements.

S4. Stamp inking procedure

First, 100 µL of NP solution is manually drop casted onto the stamp for about 2 minutes. Then, the excess of colloidal solution is removed from the side of the stamp (not from the top) with a tissue, in order to create a homogeneous layer of NPs solution on top of the stamp. If the NP solution is removed from the top, then the droplet rapidly forms a meniscus with the tissue that we use to remove the excess colloidal solution. In this case, the colloidal solution is absorbed by the tissue and the NPs tend to create large clusters at the droplet center (Figure S4a). In our case, the excess of NPs solution is removed with a tissue from the side of the stamp with several soft and fast (manual) movements until a homogeneous thin film is deposited on top of the stamp (Figure S4b).

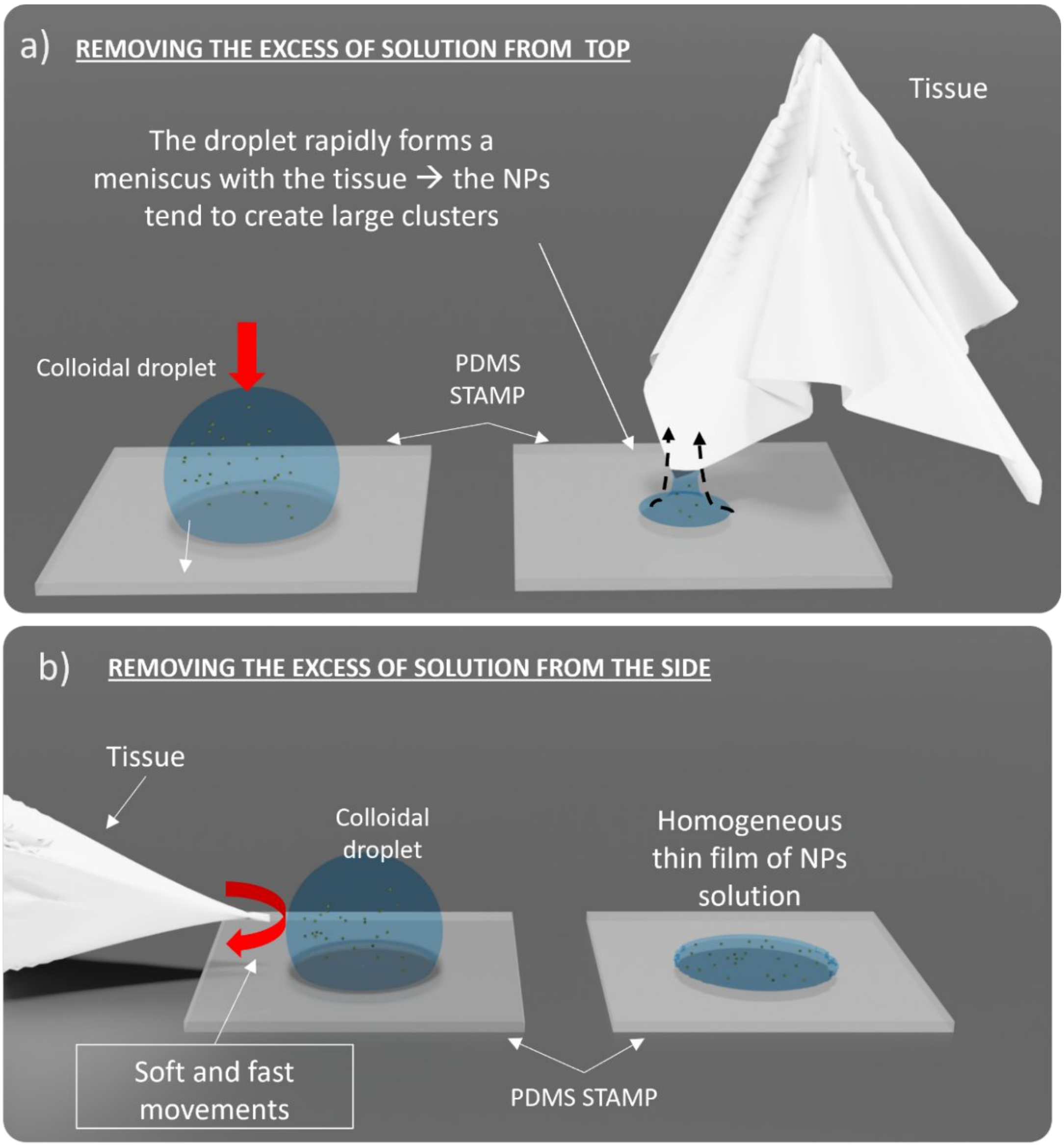

Figure S4. ***Stamp inking procedure.*** *a) Schematic view of the droplet absorbed from top. The tissue absorbs rapidly the excess of solution, and the NPs tend to create clusters at the droplet center. b) Schematic view of the droplet absorbed from the side. In this case, a homogeneous thin layer of NPs solution is deposited on top of the stamp.*

S5. Meniscus formation

Lateral optical image (Figure S5) of a macroscopic meniscus formed between a BPT functionalized gold surface and a PDMS stamp, that can serve as a macroscopic example of what we envisage is happening in the microscopic scale.

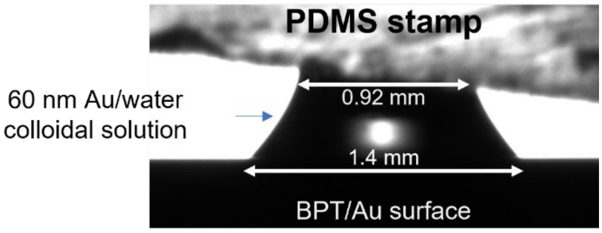

Figure S5. Lateral optical image of a macroscopic meniscus formed between a BPT functionalized gold surface and a PDMS stamp.

S6. Transfer of individual 150 nm Au NPs onto functionalized nanoantennas

Transfer of Au NPs of 150nm diameter (with a concentration of C = (2.77 ± 0.12) x 10^8 particles/ml) onto BPT functionalized samples of Au disk arrays with $W1_S$ = 1 µm stamp (Figure S6).

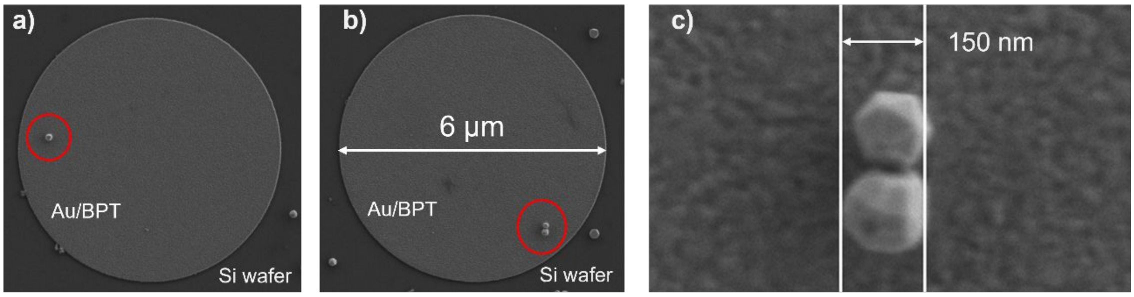

Figure S6: a) SEM image of 150 nm Au-NP transferred individually with W1S = 1 µm stamp onto a BPT/Au disk of 6µm diameter. b) SEM image of two 150 nm Au-NP transferred with W1S = 1 µm stamp onto a BPT/Au disk of 6µm diameter. c) Zoom-in of the two 150 nm Au-NPs highlighted in b).
